# Ceramide Synthase 2 Promotes Cardiac Very-Long-Chain Dihydroceramide Accumulation and Is Linked to Arrhythmias and Heart Failure in Humans

**DOI:** 10.3390/ijms26146859

**Published:** 2025-07-17

**Authors:** Linda Andersson, Mathieu Cinato, Elias Björnson, Annika Lundqvist, Azra Miljanovic, Marcus Henricsson, Per-Olof Bergh, Martin Adiels, Anders Jeppsson, Jan Borén, Malin C. Levin

**Affiliations:** 1 Department of Molecular and Clinical Medicine, Wallenberg Laboratory, Institute of Medicine, The Sahlgrenska Academy, University of Gothenburg, Sahlgrenska University Hospital, 41345 Gothenburg, Sweden; linda.andersson@wlab.gu.se (L.A.); mathieu.cinato@wlab.gu.se (M.C.); elias.bjornson@wlab.gu.se (E.B.); annika.lundqvist@wlab.gu.se (A.L.); azra.miljanovic@wlab.gu.se (A.M.); marcus.henricsson@wlab.gu.se (M.H.); per-olof.bergh@chalmers.se (P.-O.B.); martin.adiels@gu.se (M.A.); anders.jeppsson@vgregion.se (A.J.); jan.boren@wlab.gu.se (J.B.); 2 Biomarker Discovery and Development, Research and Early Development, Cardiovascular, Renal, and Metabolism (CVRM), BioPharmaceuticals R&D, AstraZeneca, 43150 Gothenburg, Sweden; 3 Department of Cardiothoracic Surgery, Sahlgrenska University Hospital, 41345 Gothenburg, Sweden

**Keywords:** dihydroceramide, ceramide synthase, hypoxia, bioactive lipids, cardiac sphingolipids

## Abstract

Acute myocardial hypoxia/ischemia is associated with abnormal accumulation of myocardial lipids, including dihydroceramides. Here, we characterized how dihydroceramides are remodeled in response to hypoxia and assessed how dihydroceramide remodeling correlates to human cardiac pathophysiology. Hypoxia resulted in a marked accumulation of very-long-chain (VLC)-dihydroceramides in cultured HL-1 cardiomyocytes. In humans, we identified a correlation between the abundance of VLC-dihydroceramides in myocardial biopsies and arrhythmias and heart failure and showed that cardiac expression of CERS2, coding for an enzyme that promotes synthesis of VLC-dihydroceramides, was associated with signaling pathways linked to cardiac arrhythmia and cardiomyopathy. In cultured HL-1 cardiomyocytes, we showed that CerS2 knockdown reduced accumulation of VLC dihydroceramides and altered the expression of mediators regulating Ca^2+^ cycling and electrical conduction. In conclusion, our findings indicate that increased abundance of VLC-dihydroceramides, promoted by increased activity of CerS2 in response to hypoxia, could play a role in cardiac arrhythmias and heart failure.

## 1. Introduction

Acute myocardial hypoxia/ischemia, a common feature of cardiovascular disease, associates with abnormal accumulation of myocardial lipids, including ceramides and their saturated analogs, dihydroceramides [1,2,3]. (Dihydro)ceramides are recognized not only as structural components of cellular membranes but also as bioactive signaling lipids, mediating differentiation, senescence and cellular stress responses [4,5]. Ceramide accumulation in the heart has been shown to be associated with cardiac dysfunction in numerous rodent models [2,3,6,7]. In humans, circulating ceramides are associated with adverse alterations in cardiac structure and function [8,9]. However, to date, there has been little focus on clarifying how (dihydro)ceramide remodeling is regulated.

Ceramides consist of a sphingoid base (most commonly 18:1) linked to a fatty acid [10]. Ceramides are synthesized de novo by a pathway that begins with the condensation of L-serine and palmitoyl CoA and involves acylation of sphinganine to dihydroceramide by ceramide synthase (CerS) [11]. Dihydroceramide is subsequently reduced to ceramide by dihydroceramide desaturase (DES) [12]. The biological activity of (dihydro)ceramides varies according to their *N*-acyl chain length [11], which is determined by the type of CerS expressed. In cardiomyocytes, very-long-chain (VLC; C20–C24) ceramides, but not long-chain (LC; C16 and C18) ceramides, induce mitochondrial dysfunction and cell death [13]. Six different enzymes (named CerS1–6) have been identified [14,15]. Their expression profiles differ between tissues, and each CerS has a specific substrate preference, leading to the synthesis of (dihydro)ceramides with different *N*-acyl chain lengths [11,16].

Earlier studies have suggested that remodeling of the sphingolipid composition may be a means for cells to cope with anoxia/hypoxia [17,18]. We have previously shown that (i) hypoxia induces an increase in dihydroceramides and ceramides in cardiomyocytes; and (ii) the increase in dihydroceramides is driven by de novo synthesis, whereas the increase in ceramides is driven by hydrolysis of sphingomyelin catalyzed by acid sphingomyelinase [3]. A previous study in mammalian cells and rat lungs showed that hypoxia promotes dihydroceramide accumulation by inactivating DES [19]. However, it remains unclear how dihydroceramide production is regulated under hypoxic conditions.

In the current study, we aimed to characterize how dihydroceramides are remodeled in response to hypoxia and to clarify how dihydroceramide profiles correlate to human cardiac pathophysiology, using cell culture models and human cardiac tissue.

## 2. Results

### 2.1. Hypoxia Induces Marked Accumulation of VLC Dihydroceramides in HL-1 Cardiomyocytes

**Figure 1:**
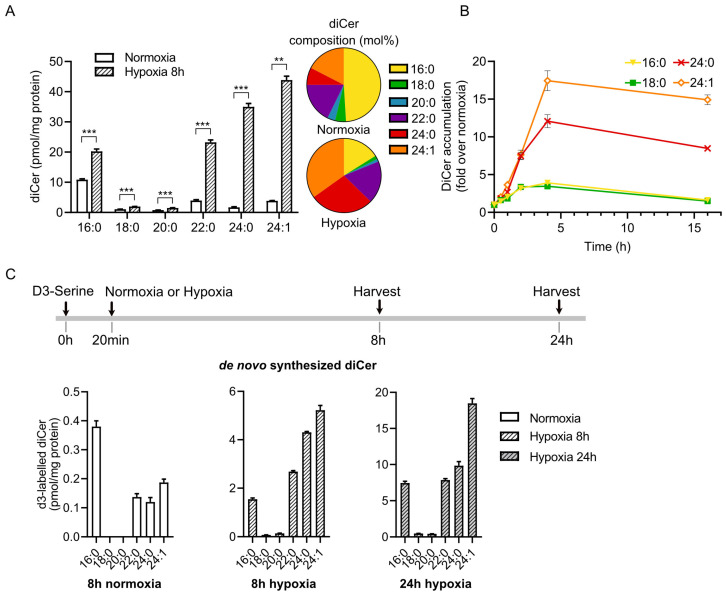
Hypoxia induces marked accumulation of VLC dihydroceramides in HL-1 cardiomyocytes. (**A**) Concentration (left) and relative abundance (right) of dihydroceramide species in HL-1 cardiomyocytes after incubation in hypoxia (1% oxygen) or normoxia for 8 h, measured by HPLC-MS (n = 3). (**B**) Change in dihydroceramide composition in hypoxia over time, measured by HPLC-MS, (n = 3). Data are shown as fold over normoxia. (**C**) Levels of de novo synthesized (d3-labelled) dihydroceramide species in HL-1 cells after incubation with d3-serine in normoxia and hypoxia for the indicated time. Data are presented as mean ± SEM. ** *p* < 0.01, *** *p* < 0.001 vs. normoxia, *t*-test.

We have previously shown that hypoxia increases accumulation of dihydroceramides in HL-1 cardiomyocytes [3]. However, intricate changes in individual dihydroceramide species under these conditions remain unknown. To investigate this, we performed a lipidomics analysis on HL-1 cardiomyocytes incubated under normoxic or hypoxic conditions. Under normoxic conditions, dihydroceramide species with *N*-acyl chain length C16:0 were the most abundant (Figure 1A). Hypoxia for 8 h induced a 1.5-fold increase in LC dihydroceramides (C16:0 and C18:0) and a dramatic (~21- and 11-fold) increase in VLC dihydroceramides (24:0 and 24:1) (Figure 1A). We further showed that accumulation of LC dihydroceramides peaked after 4 h of hypoxia, while accumulation of VLC dihydroceramides remained elevated even after 16 h of hypoxia (Figure 1B). These effects are not seen for other sphingolipid species (Figure S1).

To test if hypoxia-induced accumulation of VLC dihydroceramides resulted from an increased production rate of these lipid species, we incubated HL-1 cardiomyocytes under normoxic or hypoxic conditions in the presence of D3-serine. Under normoxic conditions, D3-serine was incorporated into C16:0 dihydroceramides to a greater extent than into the other species (Figure 1C). By contrast, under hypoxic conditions, D3-serine was incorporated into VLC dihydroceramides to a much higher extent than into LC dihydroceramides (Figure 1C). We also showed that hypoxia induced greater accumulation of VLC than LC species of the downstream metabolites dihydrosphingomyelin and dihydroglucosylceramides (Figure S2A–C). Moreover, we show that hypoxia did not alter the expression levels of Elovl1, the elongase involved in the synthesis of VLC-acyl-CoAs (Figure S3). Together, these results suggest that increased accumulation of VLC dihydroceramides under hypoxic conditions is a consequence of increased production rate rather than decreased production of VLC downstream metabolites.

### 2.2. Dihydroceramide Chain Length Correlates with Cardiac Arrhythmias and Heart Failure in Humans

**Figure 2:**
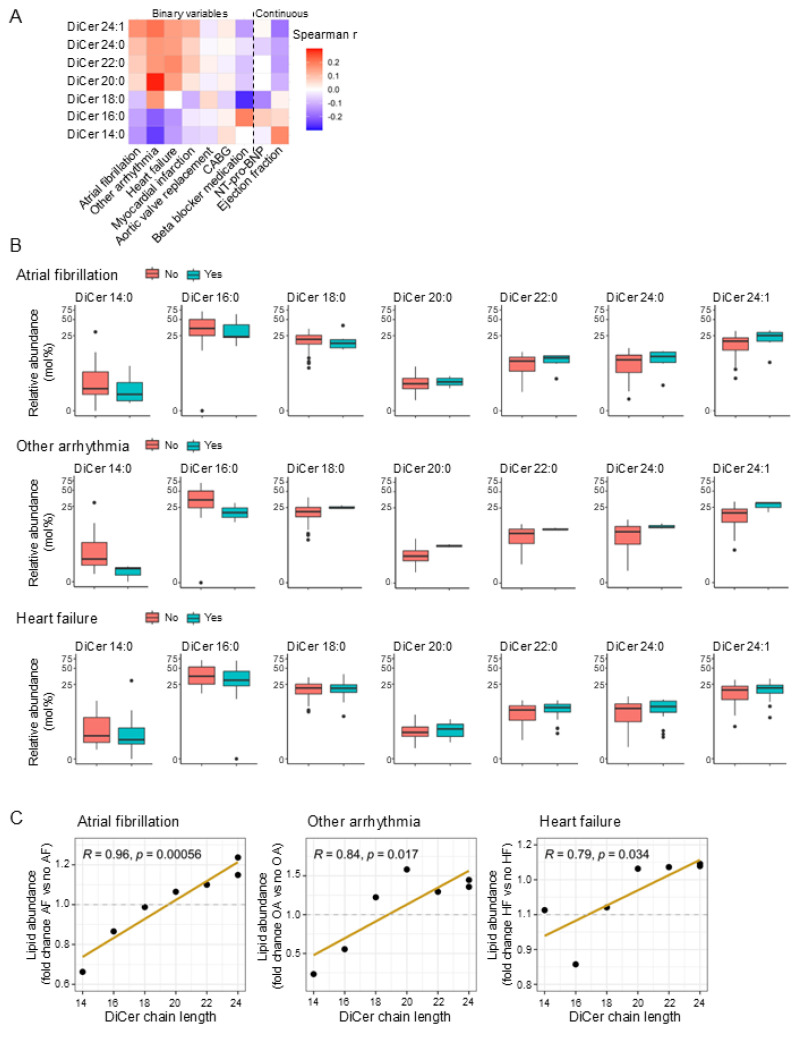
Dihydroceramide chain length correlates with cardiac arrhythmias and heart failure in humans. (**A**) Correlation matrix of the abundance of dihydroceramide species of different chain lengths in human myocardial biopsies and cardiac-related outcome variables (n = 85). (**B**) Box plots of dihydroceramide species of different chain lengths (from **A**) stratified by atrial fibrillation status, other arrythmia status and/or heart failure. Analysis is based on n = 85, of which 6 patients had AF, 3 patients had other arrhythmias, and 24 patients had heart failure. (**C**) Association between dihydroceramide chain length and the relative abundance of each lipid species in individuals with arrythmia or heart failure compared with controls. Each data point indicates one lipid species from (**A**). Correlation coefficients were calculated using the Spearman method; the variables Atrial fibrillation, Other arrhythmia, Heart failure, Myocardial infarction, Aortic valve replacement, CABG and Beta blocker medication are binary and coded as 0/1, whereas NT-pro-BNP and Ejection fraction are continuous variables.

To determine whether the marked remodeling of dihydroceramide chain length in hypoxic cardiomyocytes has potential clinical relevance, we next assessed whether the fatty acyl chain length of dihydroceramides associates with human cardiac pathophysiology. To achieve this, we examined whether the tissue abundance of specific dihydroceramide species in human myocardial tissue associates with cardiac-related clinical variables. We observed that the abundance of dihydroceramide species of increasing fatty acyl chain length correlated with arrhythmia (atrial fibrillation (AF) or other arrhythmias) and heart failure. We also found an inverse correlation between the abundance of dihydroceramide species of increasing fatty acyl chain length and the ejection fraction, but no correlations were found with other clinical variables tested (Figure 2A). There was a clear pattern of a relatively high abundance of VLC dihydroceramides and a relatively low abundance of LC dihydroceramides in the myocardial tissue of individuals with arrhythmia (atrial fibrillation or other arrhythmias) and heart failure compared to individuals without these conditions (Figure 2B). This pattern was confirmed by the presence of a positive association between chain length and relative abundance of each lipid species in individuals with atrial fibrillation, other arrhythmias and heart failure (Figure 2C).

### 2.3. High CERS2 Expression Correlates with Functional Pathways for Arrhythmogenic Cardiomyopathy, Dilated Cardiomyopathy and Hypertrophic Cardiomyopathy in Humans

**Figure 3:**
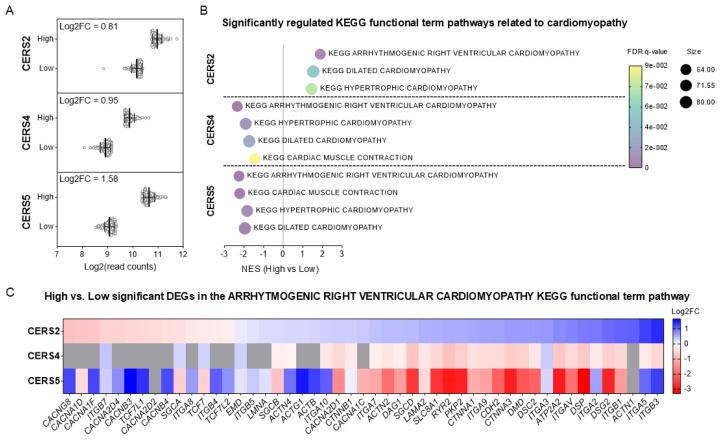
CERS2 expression in human hearts is positively associated with regulation of genes involved in arrhythmogenic cardiomyopathy. (**A**) Log2 read counts of CERS2, 4 and 5 in the left ventricle from donors with values in the bottom and top quartiles, indicating low and high expression of each gene (108 samples per group). (**B**) Significantly regulated functional terms related to cardiomyopathy from the KEGG pathway enrichment analysis of genes that were differentially expressed in heart tissues from humans with high versus low expression of each gene. (**C**) Heat map of significant differentially expressed genes (DEGs) in the arrhythmogenic right ventricular cardiomyopathy KEGG functional term pathway (49/64) from the analysis in heart tissues from humans with high versus low CERS2 expression. Log2 fold change of those genes are also shown for the analysis in heart tissues from humans with high versus low CERS4 and CERS5 expression. Gray boxes show non-significant DEGs. Source data are available for this figure.

Differences in the expression profile of CERS subtypes are known to lead to the synthesis of dihydroceramides with different *N*-acyl chain lengths [11]. To investigate whether high expression of a particular subtype of *CERS* in the human heart associates with heart function, we analyzed human RNA-seq data generated by the Genotype-Tissue Expression (GTEx) project. We extracted data on CERS2, 4 and 5 (the *CERS* that are expressed in heart [16]) in the left ventricle from 432 donors and assigned those with CERS normalized read counts in the top and bottom quantiles to high and low CERS expression groups, respectively, for each CERS subtype (Figure 3A). Kyoto Encyclopedia of Genes and Genomes (KEGG) pathway enrichment analyses of genes that were differentially expressed between the groups revealed arrhythmogenic cardiomyopathy, dilated cardiomyopathy and hypertrophic cardiomyopathy as top-ranked pathways for upregulated genes in the high CERS2 expression group (Figure 3B). Conversely, these functional term pathways were significantly ranked as downregulated genes in the high-CERS4 and -CERS5 expression groups (Figure 3B). Differentially expressed genes annotated in the arrhythmogenic cardiomyopathy pathway are shown in a heat map in Figure 3C.

### 2.4. CerS2 Depletion Reduces Hypoxia-Induced Accumulation of VLC Dihydroceramides

**Figure 4:**
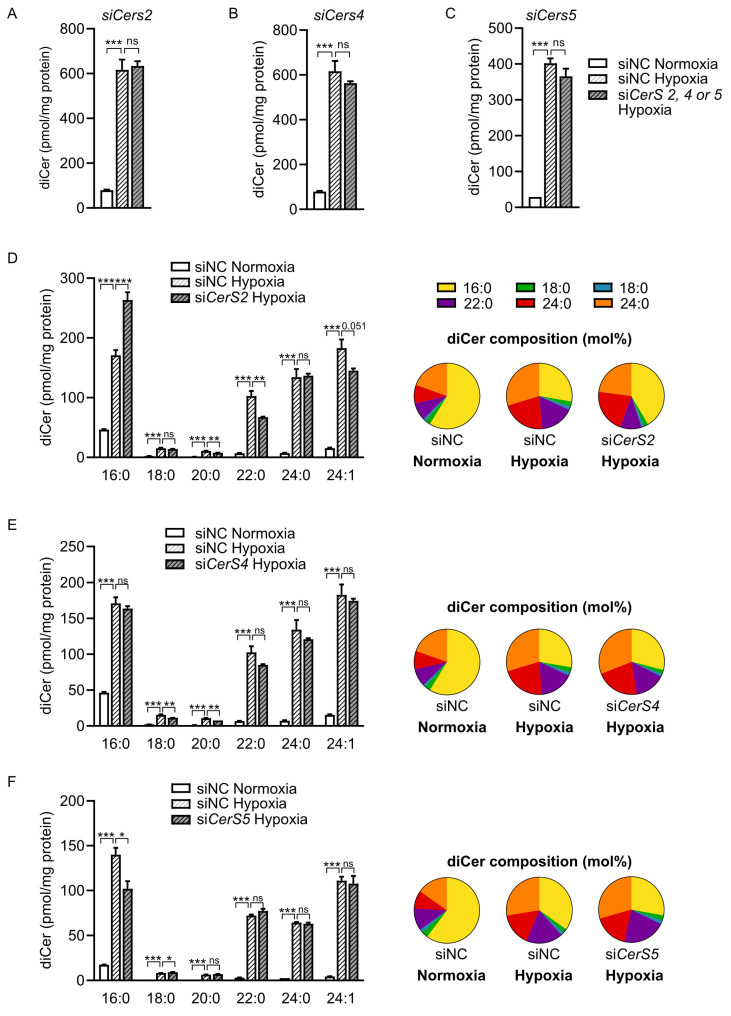
Hypoxia-induced accumulation of VLC dihydroceramides is reduced by depletion of CerS2 in HL-1 cardiomyocytes. Total concentration of dihydroceramides in HL-1 cardiomyocytes treated with (**A**) *CerS2* siRNA, (**B**) *CerS4* siRNA or (**C**) *CerS5* siRNA after incubation in hypoxia or normoxia for 8 h (**A**,**B**) or 4 h (**C**), measured by HPLC-MS (n = 3). (**D**–**F**) Concentration (left) and relative abundance (right) of dihydroceramide species in (**D**) *CerS2*-depleted, (**E**) *CerS4*-depleted and (**F**) *CerS5*-depleted HL1 cardiomyocytes after incubation in hypoxia or normoxia for 8 h (**D**,**E**) or 4 h (**F**), measured by HPLC-MS (n = 3). Data are presented as mean ± SEM, * *p* < 0.05, ** *p* < 0.01, *** *p* < 0.001, ns = not significant, one-way ANOVA followed by Tukey’s multiple comparison test.

To investigate the potential role of specific CerS subtypes in the hypoxia-induced dihydroceramide remodeling, we knocked down individual CerS subtypes (CerS2, CerS4 and CerS5, Figure S4) in HL-1 cardiomyocytes before treating the cells with hypoxia (or normoxia as control). First, we observed that the hypoxia-induced accumulation of total dihydroceramides was unaffected by depletion of either of the three subtypes (Figure 4A–C). However, depletion of CerS2 significantly altered the dihydroceramide profile following hypoxia: C16:0 was significantly increased, whereas the VLC dihydroceramides C20:0, C22:0 and C24:1, but not C24:0, were reduced (Figure 4D). By contrast, the hypoxia-induced accumulation of VLC dihydroceramides was not reduced by depletion of either CerS4 or CerS5 (Figure 4E,F). Hypoxia did not induce the mRNA expression of CerS2, CerS4 or CerS5 (Figure S5). Taken together, these results suggest that increased activity of CerS2, but not the other subtypes, could contribute to the hypoxia-induced accumulation of VLC dihydroceramides.

### 2.5. CerS2 Depletion Modulates Expression of Functional Markers in Cardiomyocytes

**Figure 5:**
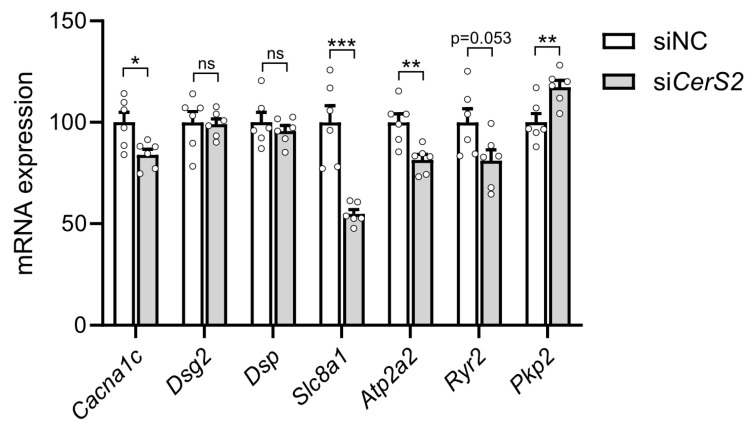
CerS2 depletion alters the expression profile of functional markers in HL-1 cardiomyocytes. mRNA expression of *Cacna1c*, *Dsg2*, *Dsp*, *Slc8a1*, *Atp2a2*, *Ryr2* and *Pkp2* in *CerS2*-deficient HL1 cardiomyocytes and in cells treated with scrambled control (siNC), 48 h after transfection with *CerS2* siRNA (n = 6). Data are presented as mean ± SEM, * *p* < 0.05, ** *p* < 0.01, *** *p* < 0.001, ns = not sigificant *t*-test.

Finally, we investigated whether modulation of CerS2 levels affects functional markers in cardiomyocytes (e.g., for cardiomyocyte contractility, calcium transporter activity, and conduction, as identified in the RNA seq analysis in Figure 3). We found that CerS2 depletion resulted in reduced levels of *Cacna1c*, *Slc8a1* and *Atp2a2* and elevated levels of *Pkp2* in HL-1 cardiomyocytes (Figure 5). These results suggest that modulation of CerS2 levels in cultured cardiomyocytes could have an effect on Ca^2+^ cycling and electrical conduction, highlighting the potential importance of CerS2 for cardiomyocyte function.

## 3. Discussion

In this study, we investigated the intracellular remodeling of dihydroceramides and explored how altered dihydroceramide composition correlates to human cardiac pathophysiology. We showed that hypoxia induced a pronounced accumulation of VLC dihydroceramides in cultured cardiomyocytes, resulting in a markedly altered dihydroceramide composition. In human hearts, we observed that VLC dihydroceramides correlated with incidence of cardiac arrhythmias and heart failure and that expression of *CERS2*, a subtype of *CERS* known to synthesize VLC dihydroceramides, was positively associated with signaling pathways linked to cardiac arrhythmia and cardiomyopathy. Subsequently, we showed that knockdown of CerS2 in cultured cardiomyocytes led to reduced accumulation of VLC dihydroceramides and to altered expression of mediators regulating Ca^2+^ cycling and electrical conduction. Taken together, our findings indicate that increased abundance of VLC dihydroceramides, promoted by increased activity of CerS2 in response to hypoxia, could play a role in cardiac arrhythmias and heart failure.

Cellular hypoxic injury predisposes humans to myocardial infarctions and arrhythmia [20,21]. Notably, cells are able to adapt the lipid profile of their membranes in response to changes in the local environment, such as hypoxia [22]. Although hypoxia-induced alteration of membrane lipid composition is regarded as a compensatory mechanism allowing organisms to survive under anoxic/hypoxic conditions [17,23], this form of stress resistance ultimately leads to cellular dysfunction. In the current study, we found that hypoxia induced a massive increase in VLC dihydroceramides in cultured HL-1 cardiomyocytes: the abundance of the VLC species 24:1 increased from 18% (of the total dihydroceramides) in normoxia to 50% in hypoxia, while the abundance of the LC species 16:0 was reduced from 50% in normoxia to less than 20% in hypoxia. Modifying the stoichiometry of certain membrane lipids has a crucial impact on membrane properties, such as membrane fluidity, and would likely affect cellular function [24]. VLC dihydroceramides have detergent-like properties [25], and may increase the propensity to membrane leakage [26]. We also observed significant correlations between cardiac VLC dihydroceramides and the incidence of arrhythmias and heart failure in humans. Given that these are disorders of cardiac electrical activity, which are closely associated with the function of cardiomyocyte membrane [27,28], it is therefore plausible that VLC dihydroceramides modify biological activities that increase the pathophysiological characteristics of arrhythmias.

The relative abundance of dihydroceramides of different chain lengths is determined by the expression profile of CERS subtypes. CERS2 is highly expressed in heart tissue and is known to catalyze the synthesis of VLC (dihydro)ceramides, ranging from C20 to C26 [11,16]. We showed that CerS2 contributes to the hypoxia-induced accumulation of VLC dihydroceramides in cultured HL-1 cardiomyocytes, indicating that hypoxia increases the activity of CerS2. The mechanisms underlying this process are currently unclear, but may involve post-translational modifications. Ceramide synthases are known to be regulated by phosphorylation [29]. In addition, CerS2 activity has been reported to be modulated by dimerization of CerS2 with other ceramide synthases or with elongases [30,31].

CERS2 has previously been associated with cardiomyocyte dysfunction in animal and cell culture models [13], and Cers2 overexpression has been shown to promote cardiotoxicity and fibrosis [32]. In humans, we showed that elevated cardiac expression of CERS2 was associated with increased expression of genes in signaling pathways linked to cardiac arrhythmia and cardiomyopathy. In particular, we observed a regulation of genes encoding multiple cardiac L-type calcium channel subunits (LTCC) (CACNA1C, CACNA2D1, CACNB1, etc.) and proteins involved in calcium cycling (RYR2, ATP2A2). Abnormal calcium handling by myocytes plays a pivotal role in the development of both contractile dysfunction and arrhythmias under pathological conditions [33]. Importantly, mutations in genes encoding the LTCC have been linked to various cardiac arrhythmias [34]. Dysregulation of calcium release from the sarcoplasmic reticulum and calcium reuptake would also be determinant for arrhythmia mechanisms directly affecting contractile force and cardiac rhythmicity [35]. Moreover, SLC8A1 (NCX1), a key transporter involved in calcium extrusion, is also decreased, potentially worsening intracellular calcium imbalances [36]. We also observe dysregulation of genes encoding proteins of the desmosome that are known to cause a subset of arrhythmogenic cardiomyopathy (PKP2, DSP, DSC2, DSG2) [37]. Indeed, in cultured HL-1 cardiomyocytes, we confirmed that Cers2 depletion reduced the expression of mediators regulating Ca^2+^ cycling and electrical conduction. Taken together, these studies indicate that lowering expression of CERS2 would potentially be beneficial by reducing the susceptibility for atrial fibrillation and arrhythmias.

This study is subject to limitations. First, the number of patients with arrhythmia, and in particular “arrhythmia other”, are very few. However, in a clinical material of human myocardial biopsies, it is challenging to include larger cohorts. Our results are strengthened by the fact that the “arrhythmia-AF” group shows the same associations as the “arrhythmia other” group. In addition, the GTEX data supports the associations shown from the clinical material. We acknowledge that the transcriptomic data from GTEx originate from an independent cohort, unrelated to the patients used for lipidomic profiling. As such, the GTEx-derived results should be interpreted as exploratory and complementary. While we could not directly validate these findings at the protein level or by qPCR due to the lack of matched tissue material, we further investigated potential mechanistic links by comparing GTEx-derived gene signatures with transcriptomic changes observed in CerS2-silenced HL-1 cells. This cross-referencing revealed that CerS2 depletion resulted in reduced levels of Cacna1c, Slc8a1, and Atp2a2, providing additional support for the involvement of CerS2 in the pathways associated with Ca^2+^ cycling and electrical conduction. Further experimental studies are now needed to support these findings and clarify the underlying mechanisms. Second, in our experimental setting studying dihydroceramide changes in hypoxia, we need to use a closed cell culture system to maintain hypoxia. For this reason, it is not possible to perform any functional assessments of cardiomyocyte function in this setting, and we need to rely on transcriptional changes as markers for function.

In conclusion, our study indicates that VLC dihydroceramide accumulation in cardiomyocytes is promoted by CerS2 activity and is associated with arrhythmias, atrial fibrillation and heart failure. Our findings thus identify the intracellular chain length of dihydroceramides as a potential pathway to target for future treatment of cardiovascular disease.

## 4. Materials and Methods

### 4.1. Cell Culture Experiments

The HL-1 cardiomyocyte cell line was a gift from W. Claycomb. The cells were cultured in supplemented Claycomb media as described previously [38] and incubated in 21% oxygen (normoxia) or 1% oxygen (hypoxia) for the indicated time.

For siRNA experiments, HL-1 cells were transfected with Silencer select siRNA targeting *CerS2* (siRNA ID:s94754), *CerS4* (siRNA ID:s205773), *CerS5* (siRNA ID:s90339), or a nontargeting negative control (4390844, Applied Biosystems, Foster City, CA, USA) using Lipofectamine RNAiMax transfection reagent (Life Technologies, Carlsbad, CA, USA) as described by the manufacturer. Cells were incubated under normoxic conditions for 40 or 44 h after transfection and thereafter under normoxic or hypoxic conditions for a further 8 or 4 h. Cells were then harvested for lipid analysis or gene expression analysis (i.e., 48 h after transfection).

For sphingolipid tracing experiments, HL-1 cardiomyocytes, at 60% confluency, were pulsed with 0.1 mg/L 2,3,3-D_3_ L-serine (Cambridge Isotope Laboratories, Andover, MA, USA) in supplemented Claycomb media [38] for 20 min and then cultured at 37 °C under normoxic or hypoxic conditions for the indicated time. Cells were then harvested for lipid analysis.

### 4.2. Human Myocardial Biopsies

Myocardial biopsies were obtained from 85 patients (16 with type 2 diabetes and 69 without type 2 diabetes) undergoing elective aortic valve replacement surgery (AVR) or coronary artery bypass graft surgery (CABG) at Sahlgrenska University Hospital, Gothenburg, Sweden. One patient underwent both AVR and CABG. Patients underwent AVR either due to aortic valve stenosis or aortic valve regurgitation. All procedures were performed with cardiopulmonary bypass (CPB) and aortic clamping. Biopsies (CABG and AVR) were analyzed as one group due to the small group size. Patient characteristics are presented in Tables S1 and S2.

Patient characteristics were either collected as the patients entered the study or retrieved later from their medical charts. The diagnoses were based on ICD-codes from patient records and national databases. Left ventricle ejection fraction (LVEF) was assessed by echocardiography preoperatively. Biopsies (10–100 mg) were taken from the auricle of the right atrium while cannulating the heart for CPB. The tissue samples were frozen immediately after harvest and then stored at −70 °C until further analysis. All patients gave written informed consent. The study protocol was approved by the Ethical Committee of the University of Gothenburg (Ethical approval code: 064-14) and performed according to the ethical guidelines of the 1975 Declaration of Helsinki.

### 4.3. Lipid Analysis

Human heart biopsies were homogenized in butanol:methanol (3:1 vol/vol) for 5 min at 25 Hz using a Mixer Mill instrument (Retch, Haan, Germany). Known amounts of internal standards were added to the samples before extraction, which was performed as described previously [39]. The lipid extracts were evaporated under nitrogen, reconstituted in chloroform–methanol (2:1 vol/vol) and stored at −20 °C until further analysis.

HL-1 cardiomyocytes were washed twice with PBS, scraped in PBS and pelleted by centrifugation for 10 min at 500× *g*. After addition of internal standard (ceramide C17:0), lipids were extracted from the cell pellet according to Lofgren et al. [39]. The lipid extracts were evaporated under nitrogen, reconstituted in chloroform–methanol (2:1 vol/vol) and stored at −20 °C until further analysis.

Before analysis, lipid extracts from human heart biopsies and HL-1 cardiomyocytes were exposed to alkaline hydrolysis in order to remove interfering phospholipids. Ceramides (dihydroceramides, ceramides and dihydroglucosylceramides from cells and human hearts were analyzed using reversed-phase UPLC (1290 Infinty, Agilent Technologies, Santa Clara, CA, USA) coupled to a QTRAP 5500 mass spectrometer (Sciex, Toronto, ON, Canada) as previously described [40]. Sphingomyelin and dihydrosphingomyelin were analyzed using direct infusion mass spectrometry according to previous work [41].

### 4.4. Analysis of Human RNA-Seq Data

Human RNA-seq data were obtained from the Genotype-Tissue Expression project. The gene read counts for the Heart–Left Ventricle RNA-Seq GTEx version 8 dataset (gene_reads_2017-06-05_v8_heart_left_ventricle.gct.gz) were downloaded from the GTEx Portal (https://gtexportal.org/home/downloads/adult-gtex/overview, accessed on 5 June 2017), along with the de-identified sample annotations (GTEx_Analysis_v8_Annotations_SubjectPhenotypesDS.tx). We then filtered the 56,200 genes by removing duplicated genes and genes for which read counts for more than 5% of the patients were equal to zero. We used the *CERS2, 4* or *5* normalized read counts to stratify the data into low and high expression groups, based on the bottom and top quartiles for each gene, respectively. Subsequently, we performed differential gene expression analysis using the DESeq2 [42] package in R (version 4.3.0). The results of differential expression analysis were then used for functional analysis. Pathway enrichment analysis of ranked gene lists was implemented using the Gene Set Enrichment Analysis (GSEA) v4.01.0 software [43]. GSEA was run using the c2.cp.kegg.v2023.1.Hs.symbols.gmt gene sets database, with phenotype permutation (1000 iterations); a GSEA false discovery rate (FDR) <0.25 was considered statistically significant.

### 4.5. Gene Expression Analysis

Total RNA was extracted from HL-1 cardiomyocytes with the RNeasy Mini Kit (Qiagen, Germantown, MD, USA) and quantified with a NanoDrop2000 spectrophotometer (Thermo Fisher Scientific, Waltham, MA, USA). cDNA was synthesized using a high-capacity cDNA Reverse Transcription Kit (Thermo Fisher Scientific Baltics, Vilnius, Lithuania), with random primers. Quantitative real-time PCR amplification of cDNA was performed using the SsoAdvanced Universal SYBR Green (Bio-Rad, Hercules, CA, USA)) or TaqMan Fast Advanced Master Mix (Thermo Fisher Scientific, Waltham, MA, USA) in a ViiA 7 Real-Time PCR system (Applied Biosystems). mRNA expression was normalized to *ACT* or *Ppia* mRNA expression using the ΔΔCT method and expressed as fold change versus controls. Sequences of primers and TaqMan references used for real-time PCR are listed in Table S3.

### 4.6. Statistical Analysis

Values are reported as means ± standard error of the mean (SEM). Details of the statistical analysis are indicated in the figure legends. An unpaired two-tailed *t* test was used for comparisons of two groups. ANOVA followed by Tukey’s multiple comparisons test (alpha 0.05) was used for comparison of more than two groups. *p* < 0.05 was considered statistically significant. Correlation coefficients in Figure 2 refer to Spearmans’s correlation coefficient, with corresponding *p*-values calculated via a two-tailed Spearman correlation test (implemented using the stat_cor() function from the *ggpubr* package in R). GraphPad Prism Software (version 10) or R (version 4.3.2) were used for all statistical analyses.

## Data Availability

This study did not generate any large-scale sequencing or proteomics datasets. This study did not generate Western blot images or microscopy data. Any additional information required to reanalyze the data reported in this paper is available from the corresponding author upon reasonable request.

## Supplementary Materials

The following supporting information can be downloaded at: https://www.mdpi.com/article/10.3390/ijms26146859/s1.

## Abbreviations

The following abbreviations are used in this manuscript:

VLC	Very-long-chain
CerS	Ceramide synthase
DES	Dihydroceramide desaturase
LC	Long-chain
LVEF	Left ventricular ejection fraction
ARV	Aortic valve replacement
CABG	Coronary artery bypass graft
CPB	Cardiopulmonary bypass
AF	Atrial fibrillation
GSEA	Gene set enrichment analysis
FDR	False discovery rate
SEM	Standard error of the mean

## References

[1] Drevinge C., Karlsson L.O., Stahlman M., Larsson T., Perman Sundelin J., Grip L., Andersson L., Boren J., Levin M.C. (2013). Cholesteryl esters accumulate in the heart in a porcine model of ischemia and reperfusion. PLoS ONE.

[2] Perman J.C., Bostrom P., Lindbom M., Lidberg U., StAhlman M., Hagg D., Lindskog H., Scharin Tang M., Omerovic E., Mattsson Hulten L. (2011). The VLDL receptor promotes lipotoxicity and increases mortality in mice following an acute myocardial infarction. J. Clin. Investig..

[3] Klevstig M., Stahlman M., Lundqvist A., Scharin Tang M., Fogelstrand P., Adiels M., Andersson L., Kolesnick R., Jeppsson A., Boren J. (2016). Targeting acid sphingomyelinase reduces cardiac ceramide accumulation in the post-ischemic heart. J. Mol. Cell. Cardiol..

[4] Bikman B.T., Summers S.A. (2011). Ceramides as modulators of cellular and whole-body metabolism. J. Clin. Investig..

[5] Summers S.A., Chaurasia B., Holland W.L. (2019). Metabolic Messengers: Ceramides. Nat. Metab..

[6] Park T.S., Hu Y., Noh H.L., Drosatos K., Okajima K., Buchanan J., Tuinei J., Homma S., Jiang X.C., Abel E.D. (2008). Ceramide is a cardiotoxin in lipotoxic cardiomyopathy. J. Lipid Res..

[7] Russo S.B., Baicu C.F., Van Laer A., Geng T., Kasiganesan H., Zile M.R., Cowart L.A. (2012). Ceramide synthase 5 mediates lipid-induced autophagy and hypertrophy in cardiomyocytes. J. Clin. Investig..

[8] Nwabuo C.C., Duncan M., Xanthakis V., Peterson L.R., Mitchell G.F., McManus D., Cheng S., Vasan R.S. (2019). Association of Circulating Ceramides With Cardiac Structure and Function in the Community: The Framingham Heart Study. J. Am. Heart Assoc..

[9] Peterson L.R., Xanthakis V., Duncan M.S., Gross S., Friedrich N., Volzke H., Felix S.B., Jiang H., Sidhu R., Nauck M. (2018). Ceramide Remodeling and Risk of Cardiovascular Events and Mortality. J. Am. Heart Assoc..

[10] Breslow D.K., Weissman J.S. (2010). Membranes in balance: Mechanisms of sphingolipid homeostasis. Mol. Cell.

[11] Zelnik I.D., Rozman B., Rosenfeld-Gur E., Ben-Dor S., Futerman A.H. (2019). A Stroll Down the CerS Lane. Adv. Exp. Med. Biol..

[12] Geeraert L., Mannaerts G.P., van Veldhoven P.P. (1997). Conversion of dihydroceramide into ceramide: Involvement of a desaturase. Biochem. J..

[13] Law B.A., Liao X., Moore K.S., Southard A., Roddy P., Ji R., Szulc Z., Bielawska A., Schulze P.C., Cowart L.A. (2018). Lipotoxic very-long-chain ceramides cause mitochondrial dysfunction, oxidative stress, and cell death in cardiomyocytes. FASEB J..

[14] Mullen T.D., Hannun Y.A., Obeid L.M. (2012). Ceramide synthases at the centre of sphingolipid metabolism and biology. Biochem. J..

[15] Pewzner-Jung Y., Ben-Dor S., Futerman A.H. (2006). When do Lasses (longevity assurance genes) become CerS (ceramide synthases)?: Insights into the regulation of ceramide synthesis. J. Biol. Chem..

[16] Levy M., Futerman A.H. (2010). Mammalian ceramide synthases. IUBMB Life.

[17] Crowder C.M. (2009). Cell biology. Ceramides--friend or foe in hypoxia?. Science.

[18] Lachkar F., Ferre P., Foufelle F., Papaioannou A. (2021). Dihydroceramides: Their emerging physiological roles and functions in cancer and metabolic diseases. Am. J. Physiol. Endocrinol. Metab..

[19] Devlin C.M., Lahm T., Hubbard W.C., Van Demark M., Wang K.C., Wu X., Bielawska A., Obeid L.M., Ivan M., Petrache I. (2011). Dihydroceramide-based response to hypoxia. J. Biol. Chem..

[20] Plant L.D., Xiong D., Romero J., Dai H., Goldstein S.A.N. (2020). Hypoxia Produces Pro-arrhythmic Late Sodium Current in Cardiac Myocytes by SUMOylation of Na(V)1.5 Channels. Cell Rep..

[21] Specterman M.J., Aziz Q., Li Y., Anderson N.A., Ojake L., Ng K.E., Thomas A.M., Finlay M.C., Schilling R.J., Lambiase P.D. (2023). Hypoxia Promotes Atrial Tachyarrhythmias via Opening of ATP-Sensitive Potassium Channels. Circ. Arrhythm. Electrophysiol..

[22] Steels E.L., Learmonth R.P., Watson K. (1994). Stress tolerance and membrane lipid unsaturation in Saccharomyces cerevisiae grown aerobically or anaerobically. Microbiology.

[23] Jain I.H., Calvo S.E., Markhard A.L., Skinner O.S., To T.L., Ast T., Mootha V.K. (2020). Genetic Screen for Cell Fitness in High or Low Oxygen Highlights Mitochondrial and Lipid Metabolism. Cell.

[24] Menuz V., Howell K.S., Gentina S., Epstein S., Riezman I., Fornallaz-Mulhauser M., Hengartner M.O., Gomez M., Riezman H., Martinou J.C. (2009). Protection of C. elegans from anoxia by HYL-2 ceramide synthase. Science.

[25] Jimenez-Rojo N., Sot J., Viguera A.R., Collado M.I., Torrecillas A., Gomez-Fernandez J.C., Goni F.M., Alonso A. (2014). Membrane permeabilization induced by sphingosine: Effect of negatively charged lipids. Biophys. J..

[26] Contreras F.X., Sot J., Alonso A., Goni F.M. (2006). Sphingosine increases the permeability of model and cell membranes. Biophys. J..

[27] Levitan I., Fang Y., Rosenhouse-Dantsker A., Romanenko V. (2010). Cholesterol and ion channels. Subcell. Biochem..

[28] Leifert W.R., Jahangiri A., McMurchie E.J. (2000). Membrane fluidity changes are associated with the antiarrhythmic effects of docosahexaenoic acid in adult rat cardiomyocytes. J. Nutr. Biochem..

[29] Sassa T., Hirayama T., Kihara A. (2016). Enzyme Activities of the Ceramide Synthases CERS2-6 Are Regulated by Phosphorylation in the C-terminal Region. J. Biol. Chem..

[30] Laviad E.L., Kelly S., Merrill A.H., Futerman A.H. (2012). Modulation of ceramide synthase activity via dimerization. J. Biol. Chem..

[31] Ohno Y., Suto S., Yamanaka M., Mizutani Y., Mitsutake S., Igarashi Y., Sassa T., Kihara A. (2010). ELOVL1 production of C24 acyl-CoAs is linked to C24 sphingolipid synthesis. Proc. Natl. Acad. Sci. USA.

[32] Kretzschmar T., Bekhite M.M., Wu J.M.F., Haase D., Forster M., Muller T., Nietzsche S., Westermann M., Franz M., Graler M.H. (2021). Long-Chain and Very Long-Chain Ceramides Mediate Doxorubicin-Induced Toxicity and Fibrosis. Int. J. Mol. Sci..

[33] Bers D.M., Despa S. (2006). Cardiac myocytes Ca2+ and Na+ regulation in normal and failing hearts. J. Pharmacol. Sci..

[34] Zhang Q., Chen J., Qin Y., Wang J., Zhou L. (2018). Mutations in voltage-gated L-type calcium channel: Implications in cardiac arrhythmia. Channels.

[35] Kistamas K., Veress R., Horvath B., Banyasz T., Nanasi P.P., Eisner D.A. (2020). Calcium Handling Defects and Cardiac Arrhythmia Syndromes. Front. Pharmacol..

[36] Scranton K., John S., Angelini M., Steccanella F., Umar S., Zhang R., Goldhaber J.I., Olcese R., Ottolia M. (2024). Cardiac function is regulated by the sodium-dependent inhibition of the sodium-calcium exchanger NCX1. Nat. Commun..

[37] Chua C.J., Morrissette-McAlmon J., Tung L., Boheler K.R. (2023). Understanding Arrhythmogenic Cardiomyopathy: Advances through the Use of Human Pluripotent Stem Cell Models. Genes.

[38] Claycomb W.C., Lanson N.A., Stallworth B.S., Egeland D.B., Delcarpio J.B., Bahinski A., Izzo N.J. (1998). HL-1 cells: A cardiac muscle cell line that contracts and retains phenotypic characteristics of the adult cardiomyocyte. Proc. Natl. Acad. Sci. USA.

[39] Lofgren L., Forsberg G.B., Stahlman M. (2016). The BUME method: A new rapid and simple chloroform-free method for total lipid extraction of animal tissue. Sci. Rep..

[40] Amrutkar M., Cansby E., Nunez-Duran E., Pirazzi C., Stahlman M., Stenfeldt E., Smith U., Boren J., Mahlapuu M. (2015). Protein kinase STK25 regulates hepatic lipid partitioning and progression of liver steatosis and NASH. FASEB J. Off. Publ. Fed. Am. Soc. Exp. Biol..

[41] Stahlman M., Fagerberg B., Adiels M., Ekroos K., Chapman J.M., Kontush A., Boren J. (2013). Dyslipidemia, but not hyperglycemia and insulin resistance, is associated with marked alterations in the HDL lipidome in type 2 diabetic subjects in the DIWA cohort: Impact on small HDL particles. Biochim. Et Biophys. Acta.

[42] Love M.I., Huber W., Anders S. (2014). Moderated estimation of fold change and dispersion for RNA-seq data with DESeq2. Genome Biol..

[43] Subramanian A., Tamayo P., Mootha V.K., Mukherjee S., Ebert B.L., Gillette M.A., Paulovich A., Pomeroy S.L., Golub T.R., Lander E.S. (2005). Gene set enrichment analysis: A knowledge-based approach for interpreting genome-wide expression profiles. Proc. Natl. Acad. Sci. USA.

