# Supplementary material for: Ceramide Synthase 2 Promotes Cardiac Very-Long-Chain Dihydroceramide Accumulation and Is Linked to Arrhythmias and Heart Failure in Humans
Source: Int J Mol Sci. 2025 Jul 17;26(14):6859. doi: 10.3390/ijms26146859 (PMC12295622; doi:10.3390/ijms26146859)
Supplement: Supplementary file 1 [file ijms-26-06859-s001.zip › ijms-3686932-supplementary.pdf]

Supplementary Material

Ceramide synthase 2 promotes cardiac very-long-chain dihydroceramide accumulation and is linked to arrhythmias and heart failure in humans

Linda Andersson[1], Mathieu Cinato[1], Elias Björnson[1], Annika Lundqvist[1], Azra Miljanovic[1], Marcus Henricsson[1,2], Per-Olof Bergh[1], Martin Adiels[1], Anders Jeppsson[1,3], Jan Borén[1] and Malin C. Levin[1#]

Corresponding author: Malin Levin (malin.levin@wlab.gu.se)

Table S1. Baseline characteristics

Variable	
n	85
Sex (% male)	62 (72.9)
Age (y)	67± 9
BMI (kg/m²)	26.1 ± 3.4
NT-proBNP (mmol/l)	354.00 [142.00, 827.00]
Creatinine (μmol/l)	82 ± 19
Treated with beta blocker (%)	65 (76.5)
Treated with RAAS-inhibitiors (%)	51 (60.0)
Hypertension (%)	51 (60.0)
Heart failure (%)	24 (28.2)
LVEF (%)	56± 10
History of MI (%)	28 (32.9)
Procedure, AVR (%)	31 (36.5)
Procedure, CABG (%)	54 (63.5)
Arrhythmia - AF (%)	6 (7.1)
Arrhythmia - other (%)	3 (3.5)

Mean ± SD for continuous variables, median with IQR for NT-proBNP and n (%) for categorical or binary variables. BMI indicates body mass index; NT-proBNP, N-terminal prohormone of brain natriuretic peptide; IQR, interquartile range; LVEF, left ventricular ejection fraction; MI, myocardial infarction; AVR, aortic valve replacement; CABG, coronary artery bypass graft surgery; AF, atrial fibrillation.

Table S2. Baseline characteristics stratified by heart failure

Variable	Heart Failure No	Heart Failure Yes	p	test
n	61	24		
Sex (% male)	42 (68.9)	20 (83.3)	0.279	
Age (y)	68 ± 9.7	67 ± 9.1	0.743	
BMI (kg/m²)	25.7 ± 3.2	27.0 ± 3.7	0.119	
NT-proBNP (mmol/l)	316.00 [134.00, 765.00]	401.50 [218.25, 1597.50]	0.226	nonnorm
Creatinine (μmol/l)	83 ± 20	81 (17)	0.694	
Treated with beta blocker (%)	45 (73.8)	20 (83.3)	0.515	
Treated with RAAS-inhibitiors (%)	38 (62.3)	13 (54.2)	0.658	
Hypertension (%)	39 (63.9)	12 (50.0)	0.350	
LVEF (%)	58 ± 9	52. ± 13	0.021	
History of MI (%)	21 (34.4)	7 (29.2)	0.835	
Procedure, AVR (%)	21 (34.4)	10 (41.7)	0.708	
Procedure, CABG (%)	40 (65.6)	14 (58.3)	0.708	
Arythmia - AF	1 (1.6)	5 (20.8)	0.008	
Arythmia - other	1 (1.6)	2 (8.3)	0.394	

Mean ± SD for continuous variables, median with IQR for NT-proBNP and n (%) for categorical or binary variables. BMI indicates body mass index; NT-proBNP, N-terminal prohormone of brain natriuretic peptide; IQR, interquartile range; LVEF, left ventricular ejection fraction; MI, myocardial infarction; AVR, aortic valve replacement; CABG, coronary artery bypass graft surgery; AF, atrial fibrillation.

Table S3. Sequences of primers or Taqman assay references used for gene expression analysis.

Gene	Primer sequences and Taqman assay references
Cacna1c	5′-ACATCCACGTCCTCACTGAAGC (Forward)<br>5′-CAGCATCCTGATCTCTCAAGGC (Reverse)
Dsg2	5′-AAGCACACTCACTTGGTTCG (Forward)<br>5′-AAGCACACTCACTTGGTTCG (Reverse)
Dsp	5′-TACACCTCAGGGCTGGAAACTC (Forward)<br>5′-GTAGTCTCCAGACCTCGTAAGC (Reverse)
Slc8a1	5′-TCCATCCAGTAGACTTCGTGAT (Forward)<br>5′-CCAAGCAATTCCTTACAGAGTGA (Reverse)
Atp2a2	5′-CTCCATCTGCTTGTCCAT (Forward)<br>5′-GCGGTTACTCCAGTATTG (Reverse)
Pkp2	5′-CAGGTGCTGAAGCAAACCAGAG (Forward)<br>5′-GACACTCTCTGTCAAGGTGAGC (Reverse)
Ppia	5′-CGCGTCTCCTTCGAGCTGTTTG (Forward)<br>5′-TGTAAAGTCACCACCCTGGCACAT (Reverse)
CerS2	Mm01258345_g1
CerS4	Mm00482658_m1
CerS5	Mm00510998_m1
Elovl1	Mm01188316_g1
ACTB	4352341E

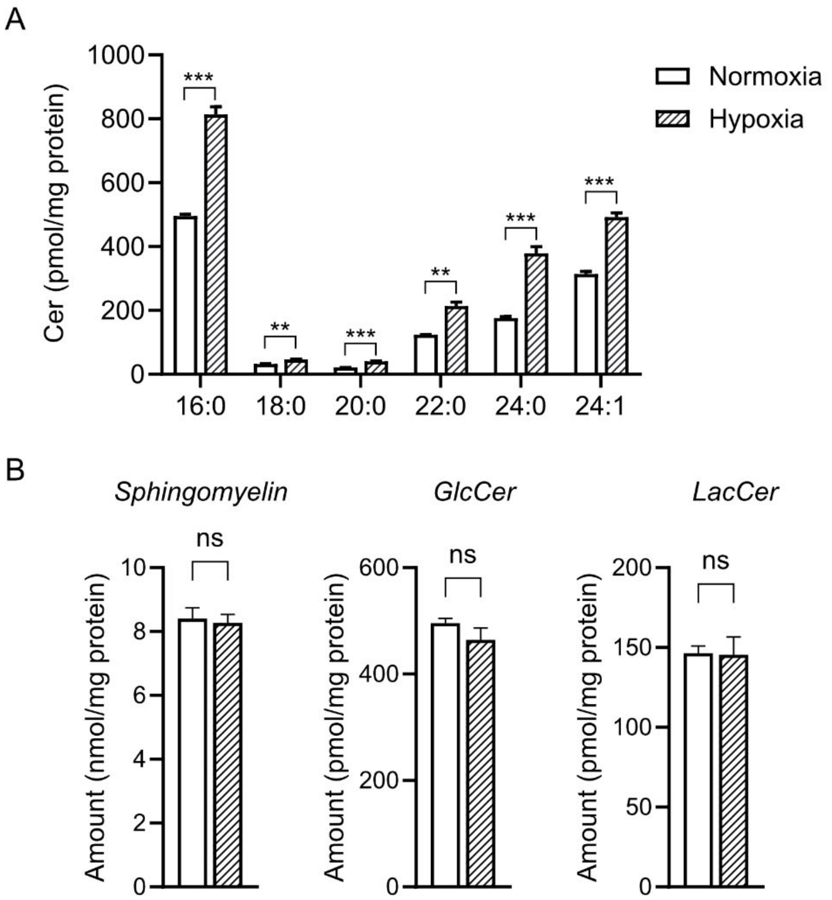

Figure S1. Hypoxia does not induces marked accumulation of VLC ceramides in HL-1 cardiomyocytes.

A) Concentration of ceramide species in HL-1 cardiomyocytes after incubation in hypoxia (1% oxygen) or normoxia for 8 h, measured by HPLC-MS (n=3). B) Total concentration of sphingomyelin, glucocylceramides (GlcCer) and lactosylceramides (LacCer) in HL-1 cardiomyocytes after incubation in hypoxia or normoxia for 8 hs, measured by HPLC-MS (n=3). Data are presented as mean ± SEM. **P<0.01, ***P<0.001 vs normoxia, t-test.

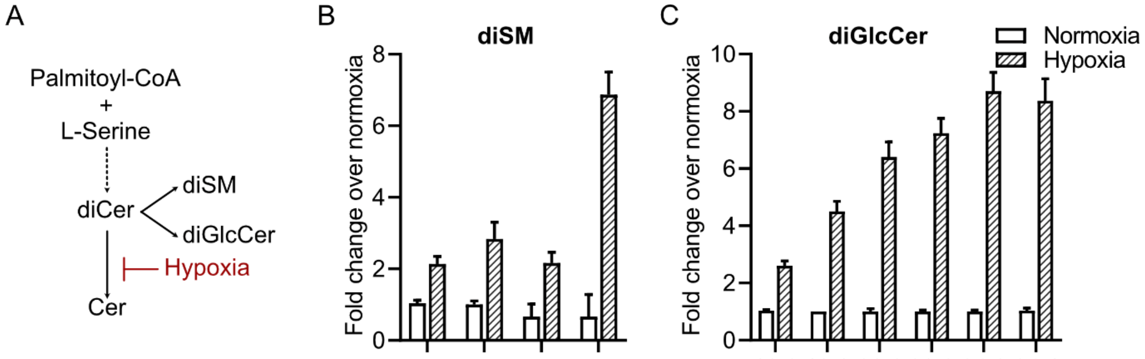

Figure S2. VLC-diSM and VLC-diGlcCer species accumulate in HL-1 cardiomyocytes during hypoxia

(A) Shematic picture of the *de novo* biosynthesis pathway of diSM and diGlcCer during hypoxia. Fold change over normoxia of diSM (B) and diGlcCer (C) species after incubation of HL-1 cardiomyocytes in normoxia or hypoxia for 8h. Values are mean +/- SEM, n=3. VLC, very long-chained, diSM, dihydrosphingomyelin; diGlcCer, dihydroglucosylceramide.

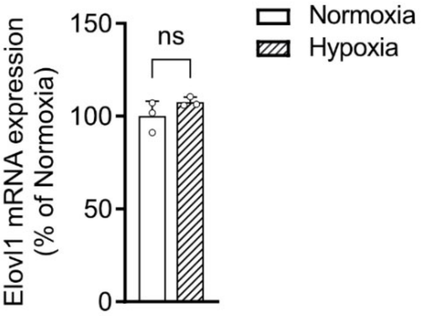

Figure S3. No change in *Elovl1* mRNA expression in HL-1 cardiomyocytes after 8h hypoxia.
mRNA expression of *Elovl1* in HL-1 cardiomyocytes after 8h hypoxia. (n=3). Values are mean ± SEM, t-test.

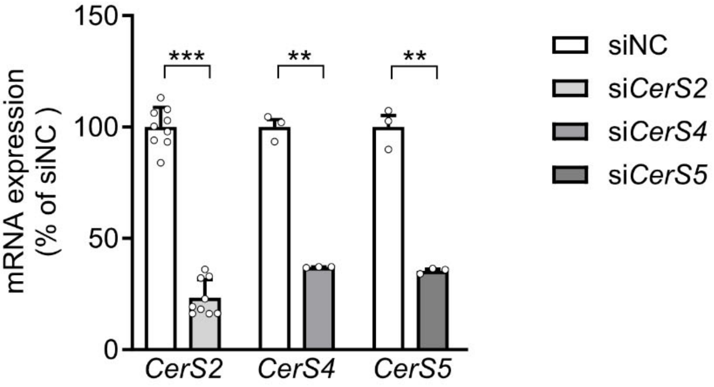

Figure S4. Validation of siRNA.
mRNA expression of *CerS2, CerS4 and CerS5* in HL-1 cardiomyocytes treated with siRNA against *CerS2*, *CerS4* or *CerS5* and scrambled control for 48 hours. (n=9 for CerS2 and n=3 for CerS4 and CerS5). Values are mean ± SEM, **P<0.01, ****P*<0.001 vs siNC, t-test.

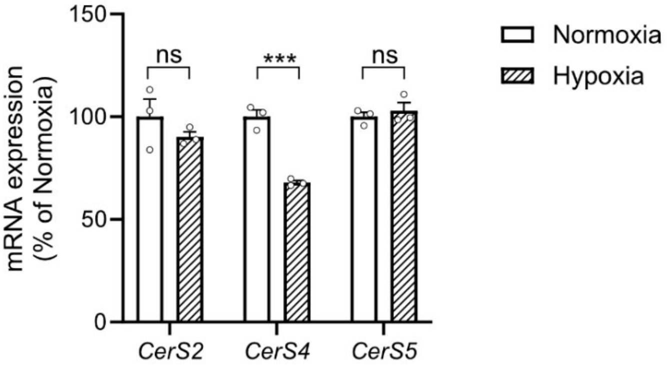

Figure S5. No **increase in mRNA levels of CerS in hypoxia**
mRNA expression of *CerS2, CerS4 and CerS5* in HL-1 cardiomyocytes after incubation in normoxia or hypoxia (1% oxygen) for 8 hours for *CerS2* and *CerS4* and 4 hours for *CerS5*. (n=3). Values are mean ± SEM, ****P*<0.001 vs normoxia, t-test.
